# Correlation between Inflammatory Markers of Atherosclerosis and Carotid Intima-Media Thickness in Obstructive Sleep Apnea

**DOI:** 10.3390/molecules19021651

**Published:** 2014-01-29

**Authors:** Marco Matteo Ciccone, Pietro Scicchitano, Annapaola Zito, Francesca Cortese, Barbara Boninfante, Vito Antonio Falcone, Vitaliano Nicola Quaranta, Valentina Anna Ventura, Antonietta Zucano, Francesca Di Serio, Mario Francesco Damiani, Onofrio Resta

**Affiliations:** 1 Cardiovascular Diseases Section, Department of Emergency and Organ Transplantation (DETO), University of Bari, Bari 70124, Italy; E-Mails: piero.sc@hotmail.it (P.S.); annapaolazito@yahoo.it (A.Z.); francescacortese@hotmail.it (F.C.).; 2 Institute of Respiratory Disease, University of Bari, Bari 70124, Italy; E-Mails: b.boninfante@alice.it (B.B.); iposoli9@gmail.com (V.A.F.); vitaliano40@libero.it (V.N.Q.); valevent@libero.it (V.A.V.); mariodmn84@hotmail.com (M.F.D.); onofrio.resta@uniba.it (O.R.).; 3 Section of Clinical Pathology, University of Bari, Bari 70124, Italy; E-Mails: azucano@libero.it (A.Z.); diseriofrancesca@tiscali.it (F.D.S.)

**Keywords:** carotid intima-media thickness, obstructive sleep apnea, high sensitive C-reactive protein, interleukin-6, tumor necrosis factor-alpha, pentraxin-3

## Abstract

Obstructive Sleep Apnea (OSA) is a sleep-related breathing disorder associated with the development of cardiovascular diseases and atherosclerosis. Systemic inflammation plays an important role in the development of cardiovascular complications in OSA patients. The aim of the study was to evaluate the relationship between carotid intima-media thickness (cIMT) and inflammatory markers plasma levels in OSA patients. We enrolled 80 OSA patients and 40 controls matched for age and body mass index (BMI). The presence and severity of sleep apnea was determined by in-laboratory portable monitoring (PM). Demographic data, blood pressure, heart rate, and cIMT were measured. High-sensitive C-Reactive Protein (hsCRP), interleukin (IL)-6, tumor necrosis factor (TNF)-α and pentraxin (PTX)-3 serum concentrations were detected. cIMT was higher in OSA patients than controls (0.89 ± 0.13 mm *vs.* 0.65 ± 0.1 mm, *p* < 0.01). Moderate-severe OSA patients (0.95 ± 0.09 mm) had significantly increased cIMT than mild OSA (0.76 ± 0.1 mm; *p* < 0.01) and control (0.65 ± 0.1 mm; *p* < 0.01). hsCRP, IL-6, TNF-α, and PTX-3 in patients with OSA (1.67 ± 0.66 mg/L, 2.86 ± 1.39 pg/mL, 20.09 ± 5.39 pg/mL, 2.1 ± 0.59 ng/mL, respectively) were significantly higher than in controls (1.08 ± 0.53 mg/L, *p* < 0.01; 1.5 ± 0.67 pg/mL, *p* < 0.01; 12.53 ± 3.48 pg/mL, *p* < 0.01; 1.45 ± 0.41 ng/mL, *p* < 0.01, respectively). Carotid IMT was significantly correlated to CRP (r = 0.44; *p* < 0.01), IL-6 (r = 0.42; *p* < 0.01), TNF-α (r = 0.53; *p* < 0.01), and PTX-3 (r = 0.49; *p* < 0.01). OSA patients showed increased cIMT, CRP, IL-6, TNF-α, and PTX-3 levels. Inflammatory markers levels are correlated to cIMT in OSA patients.

## 1. Introduction

Obstructive Sleep Apnea (OSA) is a common sleep-related breathing disorder [1], affecting 4% of middle-aged (30–60 years) males and 2% of middle-aged (30–60 years) females [2]. The disorder is characterized by recurrent episodes of upper airway obstruction during sleep [3], resulting in chronic intermittent hypoxia, sleep fragmentation, and daytime sleepiness [4,5,6]. This medical condition is associated with several cardiovascular disturbances, such as congestive heart failure, hypertension, atrial fibrillation, nocturnal arrhythmias, stroke, pulmonary hypertension, and atherosclerosis [7,8,9]. Carotid intima-media thickness (cIMT) is a useful marker of early atherosclerosis development [10]. Several studies showed a strong correlation between cIMT and risk of cardio- and cerebrovascular diseases [11,12,13,14]. In addition, an increase in cIMT in OSA patients and in otherwise healthy individuals has been widely demonstrated [15,16,17,18,19,20]; our group also demonstrated a correlation between OSA duration and cIMT [21]. On the other hand, there is increasing evidence that systemic inflammation plays an important role in the development of cardiovascular complications in OSA patients [22]. Studies demonstrated that increased serum levels of C-reactive protein (CRP), interleukin (IL)-6, tumor necrosis factor (TNF)-α, and pentraxin (PTX)-3 are important risk factors for atherosclerosis, and cardiovascular diseases [23,24,25,26]. Moreover, other studies found increased serum levels of the above mentioned inflammatory markers among OSA patients [27,28,29]. However, the relationship between inflammatory markers of atherosclerosis and cIMT has not been widely studied in OSA patients. The aims of this study were thus to evaluate levels of inflammatory markers of atherosclerosis, such as IL-6, TNF-α, CRP, and PTX-3 in a sample of OSA patients; to assess the correlation between carotid IMT and levels of these inflammatory markers.

## 2. Results

Among the 80 OSA patients, 26 had mild OSA, and 54 had moderate-severe OSA. Characteristics of the patients with OSA and control subjects, including age, sex, body mass index, neck circumference, systolic/diastolic blood pressure, heart rate, Epworth Sleepiness Scale score, apnea-hypopnea index, total sleep time with oxyhemoglobin saturation below 90% (TST90), mean arterial oxygen saturation (SaO2), and SaO2 nadir are listed in Table 1. ESS scores, AHI, and TST90 were significantly higher both in mild and moderate-severe OSA patients than in control subjects. In addition, AHI and TST90 were significantly higher in moderate-severe OSA group than in mild OSA group. Mean SaO2 and SaO2 nadir in moderate-severe OSA group were significantly lower than in mild OSA or control group. Moreover, mean SaO2 and SaO2 nadir in mild OSA patients were significantly lower than in control subjects.

**Table 1:** Demographic and polygraphic characteristics of study population.

	Controls	Mild OSA	Moderate-Severe OSA
Number	40	26	54
Sex (male/female)	34/6	23/3	45/9
Age (years)	52.27 ± 10.52	53.65 ± 11.47	52.33 ± 10.19
BMI (kg/m^2^)	28.24 ± 2.7	28.13 ± 3.04	28.8 ± 3.03
Neck-circumference (cm)	40.12 ± 2.91	40.03 ± 3.07	40.61 ± 3.41
Blood pressure (mmHg)			
- Systolic	124.37 ± 11.21	124.61 ± 10.94	127.59 ± 10.12
- Diastolic	78.62 ± 9.53	77.3 ± 9.92	80.18 ± 9.8
Heart rate (bpm)	72.3 ± 10.65	73.84 ± 11.04	73.03 ± 10.8
ESS	6.72 ± 4.03	10.03 ± 4.16*^ a^*	11.25 ± 4.81*^ c^*
AHI (events/h)	2.11 ± 1.14	10.55 ± 3.14*^ a^*	45.13 ± 16.08*^ b,c^*
TST90 (%)	0.04 ± 0.08	4.81 ± 5.24*^ a^*	29.17 ± 13.32 *^b,c^*
Mean SaO2 (%)	94.55 ± 2.05	91.92 ± 1.71*^ a^*	89.25 ± 2.78*^ b,c^*
SaO2 nadir (%)	88.1 ± 4.21	81.8 ± 6.54*^ a^*	74.35 ± 9.15*^ b,c^*

Data are presented as mean values ± standard deviation or as number. AHI: apnea-hypopnea index; BMI: body mass index; ESS: Epworth Sleepiness Scale; OSA: obstructive sleep apnea; SaO2: arterial oxygen saturation; TST90: total sleep time with oxyhemoglobin saturation below 90%. *^a^* *p* < 0.01 mild OSA *vs*. controls. *^b^* *p* < 0.01 moderate-severe OSA *vs.* mild OSA. *^c^* *p* < 0.01 moderate-severe OSA *vs*. controls.

Carotid IMT was significantly elevated in patients with OSA compared to non-OSA subjects (0.89 ± 0.13 mm *vs.* 0.65 ± 0.1 mm, *p* < 0.01). In addition, cIMT in patients with moderate-severe OSA (0.95 ± 0.09 mm) was significantly increased as compared to patients with mild OSA (0.76 ± 0.1 mm; *p* < 0.01) or to control subjects (0.65 ± 0.1 mm; *p* < 0.01).

Serum levels of CRP, and plasma levels of IL-6, TNF-α, and PTX-3 are shown in Table 2. Levels of hsCRP, IL-6, TNF-α, and PTX-3 in patients with OSA (1.67 ± 0.66 mg/L, 2.86 ± 1.39 pg/mL, 20.09 ± 5.39 pg/mL, 2.1 ± 0.59 ng/mL, respectively) were significantly higher than controls (1.08 ± 0.53 mg/L, *p* < 0.01; 1.5 ± 0.67 pg/mL, *p* < 0.01; 12.53 ± 3.48 pg/mL, *p* < 0.01; 1.45 ± 0.41 ng/mL, *p* < 0.01, respectively). Moreover, levels of hsCRP, IL-6, TNF-α, and PTX-3 in moderate-severe OSA subjects were significantly increased as compared to mild OSA patients (*p* < 0.01) or to non-OSA subjects (*p* < 0.01) (Table 2).

**Figure 1:**
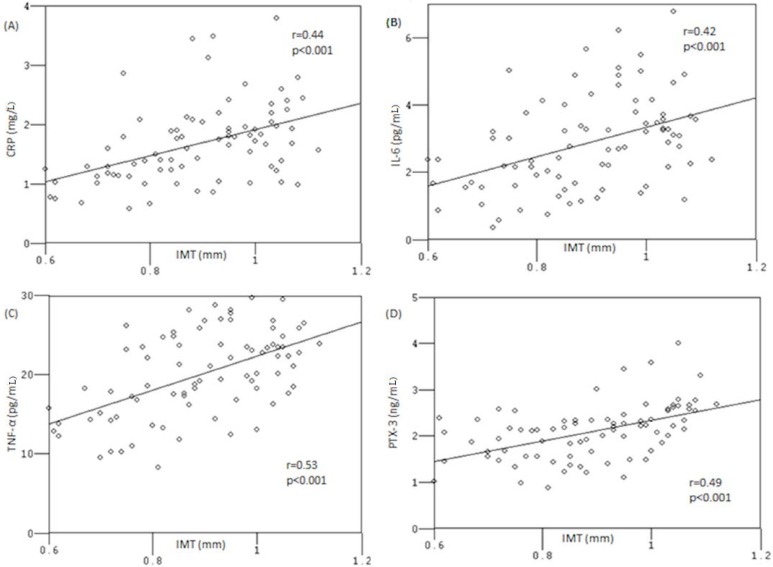
Correlation between carotid intima-media thickness (IMT) and levels of C-reactive protein [CRP] (**A**), interleukin [IL]-6 (**B**), tumor necrosis factor [TNF]-α (**C**), and pentraxin [PTX]-3 (**D**).

The correlations between cIMT and levels of inflammatory markers in OSA patients are shown in Figure 1. Carotid IMT was significantly correlated to hsCRP (r = 0.44; *p* < 0.01), IL-6 (r = 0.42; *p* < 0.01), TNF-α (r = 0.53; *p* < 0.01), and PTX-3 (r = 0.49; *p* < 0.01). We performed a regression analysis in order to evaluate the independent predictors of carotid intima-media thickness measurements (Table 3). After the statistical evaluations, we confirmed hsCRP and TNF-α as independent predictors for carotid IMT as well as PTX-3.

**Table 2:** Comparison of hsCRP, IL-6, TNF-α, and PTX-3 between groups.

	Controls	Mild OSA	Moderate-severe OSA
CRP, mg/L	1.08 ± 0.53	1.32 ± 0.48	1.84 ± 0.67*^ a,b^*
IL-6, pg/mL	1.5 ± 0.67	1.89 ± 0.88	3.33 ± 1.35*^ a,b^*
TNF-α, pg/mL	12.53 ± 3.48	14.42 ± 3.29	22.83 ± 3.85*^ a,b^*
PTX-3, ng/mL	1.45 ± 0.41	1.66 ± 0.46	2.31 ± 0.53*^ a,b^*

Data are presented as mean values ± standard deviation. hsCRP: high sensitive C-reactive protein; IL-6: interleukin-6; OSA: obstructive sleep apnea; PTX-3: pentraxin-3; TNF-α: tumor necrosis factor-α. *^a^* *p* < 0.01 moderate-severe OSA *vs.* mild OSA. *^b^ p* < 0.01 moderate-severe OSA *vs*. controls

**Table 3:** Multivariate regression analysis to evaluate the independent predictors of carotid intima-media thickness measurements.

	Β-coefficient	Standard Deviation	*p*
hsCRP, mg/L	−0.234	0.107	0.031
IL-6, pg/mL	0.130	0.099	0.194
TNF-α, pg/mL	0.027	0.013	0.041
PTX-3, ng/mL	0.863	0.047	0.000

hsCRP: high sensitive C-reactive protein; IL-6: interleukin-6; OSA: obstructive sleep apnea; PTX-3: pentraxin-3; TNF-α: tumor necrosis factor-α.

## 3. Discussion

This study demonstrated that cIMT, and hsCRP, IL-6, TNF-α, and PTX-3 were significantly higher in OSA patients than in control subjects. In addition, cIMT was significantly correlated with levels of the above-mentioned inflammatory markers.

It is widely accepted that OSA can directly worsen the endothelial function [30,31,32]. Several studies showed that OSA is responsible for increased cIMT [15,16,17,18,33]. The present study confirmed these findings. The two primary mechanisms underlying endothelial abnormalities in OSA are represented by repetitive episodes of hypoxia/reoxygenation associated with transient cessation of breath during sleep, and sleep fragmentation/deprivation [34]. These two mechanisms lead to the onset of systemic inflammation, characterized by increased levels of several inflammatory markers [34]. Ongoing inflammatory responses play an important role in atherosclerosis [35].

Some epidemiological studies suggest that hsCRP is an important risk factor for atherosclerosis, and coronary artery disease [36,37]. In addition, similarly to our study, several works have demonstrated increased hsCRP values among OSA patients [28]. However, to date, only one paper assessed the presence of a correlation between hsCRP and cIMT, in a sample of OSA subjects, wherein a significant correlation between these two parameters was found [38]; therefore, our results corroborated their findings that increased levels of hsCRP may be involved in the progression of atherosclerosis in OSA patients. Possible mechanisms of endothelial dysfunction with increased levels of hsCRP have been previously described: hsCRP is found in atherosclerotic plaque [39], it has a direct role in the secretion of inflammatory mediators from vascular endothelium [40], it promotes the expression of adhesion molecules [41], and opsonizes low-density lipoproteins for uptake by macrophages in atherosclerotic plaque [42].

Interleukin-6 is a proinflammatory cytokine which has an important role in the pathogenesis of atherosclerosis [43]. Indeed, IL-6 is an inducer of the hepatic acute phase response, stimulating the production of reagents, such as CRP, and serum amyloid A [43]. The effects of increased hsCRP values have been mentioned above; besides, there is evidence that serum amyloid A lowers high-density lipoprotein levels [43]. In addition, IL-6 is responsible for increased lipids uptake by macrophages [43]. The presence of increased levels of IL-6 in OSA patients was largely demonstrated [27]. Moreover, recently, it was found a significant correlation between IL-6, and cIMT in patients with OSA [38], such as in our work.

Several studies have showed a link between increased TNF-α values, and cardiovascular co-morbidities [25]. TNF-α stimulates monocytes adhesion to endothelial cells surface, the infiltration of the vascular wall, and their conversion to macrophages [44]. Besides, it stimulates the production of cytokines involved in atherogenesis [43]. To date, there is evidence that patients with OSA have increased TNF-α values [27]; differently, the correlation between this cytokine and a marker of early atherosclerosis, such as cIMT, has still not been evaluated among OSA subjects. Therefore, our study was the first to demonstrate the presence of a significant correlation between TNF-α, and the progression of atherosclerosis, in a sample of OSA patients.

Pentraxin-3 is produced, in response to proinflammatory signals, by diverse cell types, predominantly macrophages, and vascular endothelial cells [29]. On that account, PTX-3 is considered to be specific for endothelial inflammation [45]. To our knowledge, in literature is reported only one study in which PTX-3 levels have been evaluated in OSA patients [29]; Kasai *et al.* [29] conducted a study with 50 OSA patients, and 25 controls, and found increased PTX-3 values in patients with OSA. In this regard, our results confirmed these findings; moreover, we found a significant correlation between PTX-3, and cIMT. However, the role of PTX-3 in pathogenesis of vascular pathology is still debated [46]. On one hand, it has been demonstrated that plasma levels predict 3-month mortality in patients with myocardial infarction, even after adjustment for major risk factors and other acute-phase prognostic markers [47]. In another work, Napoleone *et al.* [48] showed that PTX-3, by up-regulating endothelial cell tissue factor, potentially plays a role in thrombogenesis. These results suggest a pathogenic role of PTX-3 in atherosclerosis. On the other hand, other studies showed an atheroprotective role of PTX-3; in this regard, it was observed that the pro-atherogenic cytokine interferon-γ inhibits, whereas the anti-atherogenic interleukin-10 stimulates the expression of PTX-3 in dendritic cells, and monocytes [49]. Furthermore, there is evidence that PTX-3 enhances apoptotic cells clearance, and therefore contributes to reduce the size of atherosclerotic lesions [50]; indeed, it is largely known that an efficient apoptotic cells clearance limits the development of atherosclerosis [51]. The present study does not allow to elucidate the role of PTX-3 in atherosclerosis; nevertheless, the presence of a significant correlation with carotid intima-media thickness suggest that PTX-3 can be considered as a marker of vascular damage in OSA subjects.

An important limitation of the present study is that sleep parameters were obtained in absence of objective sleep time; although this limit is insurmountable due to the obvious impossibility to measure these parameters in absence of electrophysiological signals, we calculated the polygraphic indices following the recommended method for cardiorespiratory monitoring [52].

## 4. Experimental

### 4.1. Patients and Study Design

We enrolled 80 subjects suffering from newly diagnosed OSA (apnea hypopnea index [AHI] ≥ 5), and 40 controls subjects not suffering from OSA (AHI < 5). Each control subject was matched for age and body mass index (BMI) to two OSA cases; matching criteria were age within ±5 years, and BMI within 2.5 kg/m^2^. The presence and severity of sleep apnea was determined by in-laboratory portable monitoring (PM). Exclusion criteria were as follows: chronic obstructive pulmonary disease, history of smoking, congestive heart failure, hypertension (blood pressure ≥ 140/90 or receiving medications), previous myocardial infarction, unstable angina, prior coronary intervention, arrhythmias, use of cardioactive drugs, chronic renal disease, diabetes mellitus, morbid obesity (BMI > 40 kg/m^2^), any chronic inflammatory disease, and systemic infections at the time of the study or within two weeks before the study. Demographic data, blood pressure, heart rate, and cIMT were measured during the morning before PM; blood samples were collected in the morning (between 7 AM and 8 AM) after PM.

The study was approved by the Institutional Review Board of Bari University General Hospital and carried out in accordance with the principles of the Helsinki Declaration. All patients gave prior written informed consent to take part in the study.

### 4.2. Portable Monitoring

The portable monitor used in the laboratory (SomtèCompumedics Inc., Abbotsford, VIC, Australia) recorded the following signals: nasal airflow, thoracic and abdominal movements, arterial oxygen saturation, electrocardiogram, body position, and snoring. PM recordings were interpreted by two sleep medicine physicians according to recommendations provided by the American Academy of Sleep Medicine [52]. An apnea-hypopnea index ≥5 was necessary to diagnose OSA; an AHI of ≥5 to <15 indicated mild OSA, and an AHI ≥15 indicated moderate-severe OSA. Sleep time was obtained as follows: each patient filled out a sleep diary, and hours in which patients reported they had not slept were subtracted from total hours of recording time. Outpatient sleep parameters were derived from the presumed sleep time (or useful recording time) [53]. PM recordings were not considered reliable if the quality of one of the main signals (oxygen saturation, thoracic/abdominal movements, nasal airflow) was poor for more than 20% of the recording time. The Epworth Sleepiness Scale was performed to assess daytime sleepiness.

### 4.3. Carotid IMT Assessment

All patients underwent two-dimensional echo-color Doppler of the carotid arteries, adopting a Philips Sonos 5500 (Bothell, WA, USA) high definition vascular echograph and a 10-3 MHz linear electronic probe. All the examinations were performed by the same physician. During the procedure, patients were placed in supine position, with the neck extended and turned contralaterally by about 45°. The cIMT was defined as the distance between the lumen intima and media-adventitia borders of the vessel, ultrasonographically identified by a double hypoechoic line not projecting into the vessel lumen [54,55]. The intima-media thickness of the distal wall of the right common carotid artery on the lengthwise axis, was calculated according to the method described by Pignoli *et al*. [56]. Echo-measurements were made in three zones: (a) proximal zone: about 2 cm before the flow-divider; (b) distal zone: about ½ cm before the flow-divider; (c) middle zone. These data were used to obtain the arithmetical mean cIMT value.

### 4.4. Measurement of Inflammatory Markers

Samples of peripheral venous blood were collected between 7 AM and 8 AM. Samples were stored at −80 °C until the time of assay. hsCRP concentration was measured using a latex-particle enhanced turbidimetric immunoassay. Quantitative sandwich enzyme immunoassay kits (R&D Systems, Minneapolis, MN, USA) were used to measure IL-6, TNF-a and PTX-3 concentrations in plasma.

### 4.5. Statistical Analysis

Data are presented as mean ± SD unless otherwise indicated. Differences between two groups wereanalyzed by a Student’s t-test for independent samples. Differences between three groups wereanalyzed by analysis of variance with Bonferroni correction. Correlation were described with the Pearson correlation coefficient (r). A value of *p* < 0.05 was considered statistically significant. The analyses were made using STATISTICA 6.1 software (StatSoft Inc., Tulsa, OK, USA).

## 5. Conclusions

We have demonstrated that OSA patients have increased levels of cIMT, hsCRP, IL-6, TNF-α, and PTX-3. These inflammatory markers may have a role in the progression of atherosclerosis among OSA subjects. Thus, we found a correlation between these inflammatory markers, and cIMT, in a sample of OSA patients. In particular, hsCRP, TNF-α, and PTX-3 are independent predictors of cIMT values.
